# Microscopic research on the olfactory organ of the Far Eastern brook lamprey *Lethenteron reissneri* (Pisces, Petromyzontidae)

**DOI:** 10.1186/s42649-020-00038-3

**Published:** 2020-09-15

**Authors:** Hyun-Tae Kim, Jong-Young Park

**Affiliations:** Department of Biological Science and Institute for Biodiversity Research, College of Natural Sciences, Jeonbuk National University, Jeonju, 54896 South Korea

**Keywords:** Ciliary length lamellae, Olfactory receptor neuron, Single olfactory organ, Tube nostril

## Abstract

The olfactory anatomy and histology of *Lethenteron reissneri* were researched using a stereo microscope, a light microscope, and a scanning electron microscope. As in other lampreys, it shows same characters as follows: i) a single olfactory organ, ii) a single tubular nostril, iii) a single olfactory chamber with gourd-like form, iv) a nasal valve, v) a nasopharyngeal pouch, vi) a sensory epithelium (SE) of continuous distribution, vii) a supporting cells with numerous long cilia, viii) an accessory olfactory organ. However, the description of a pseudostratified columnar layer in the SE and Non SE is a first record, not reported in sea lamprey *Petromyzon marinus*. In particular, both 19 to 20 lamellae in number and olfactory receptor neuron’s quarter ciliary length of the knob diameter differ from those of *P. marinus*. From these results, it might be considered that the olfactory organ of *L. reissneri* shows well adaptive structure of a primitive fish to slow flowing water with gravel, pebbles, and sand and a hiding habit into sand bottom at daytime. The lamellar number and neuron’s ciliary length may be a meaningful taxonomic character for the class Petromyzonida.

## Introduction

In fishes, the olfactory organ is an essential chemoreceptor that processes smells and deeply related to diverse ecological behaviors: prey detection, feeding, reproduction, predator risk avoidance, home recognition and migration (Hara 1986). This organ also is a good morphological indicator to reflect aquatic habitat conditions: i) a degree of water volume, a velocity, a turbidity, and a bottom structure as physical factors (Kim et al. 2019) and ii) pH, infective pathogens, hazard materials, and types of chemical odors as chemical factors (Ghosh and Mandal 2014) and iii) fish’s ecology (Yamamoto 1982). Finally, the above factors may lead the olfactory organ to have some modification in morphology, rosette structure, lamellar number and arrangement, and sensory epithelial distribution (Yamamoto 1982; Zeiske et al. 1992; Kasumyan 2004). So far, fish’s olfactory researches have mainly focused on the higher vertebrate group. The lower lineage, especially the cyclostomata, has not been known well (Kasumyan 2004; Ferrando et al. 2019; Ren et al. 2009).

The cyclostomata is the most primitive vertebrate, and consist of the two classes, Petromyzonida (lamprey) and Myxini (hagfish) (Yang et al. 2016). Among them, lamprey retains strong resemblance to a fossil organism that has early vertebrate characters (Gess et al. 2006), so that they have been highlighted as a best model for studying the embryonic development, evolution, and organ differentiation of fish taxa (Yang et al. 2016). Lamprey has currently recognized 38 species (Docker 2006), most of which are parasitic and carnivorous to feed blood other fishes and migrate from saltwater to freshwater (Hardisty 2013). Some are non-carnivorous species to live exclusively in freshwater (Potter et al. 2015). Meanwhile, anadromous and landlocked Atlantic salmon showed physiological and histological differences in acclimation to seawater, i.e. the anadromous fish has larger chloride cells of the gill in size and more activated Na^+^, K^+^ ATPase than landlocked fish (Nilsen et al. 2007). So far, the olfactory research of lamprey, however, has only been focused on anadromous saltwater species (Kleerekoper and Erkel 1960; Van Denbossche et al. 1995, 1997; Ren et al. 2009). With the non-anadromous and landlocked fish, *Lethenteron reissneri*, therefore, anatomical and histological studies on the olfactory organ was carried out, particularly with regard to ecological adaptation.

## Materials and methods

### Specimen preparation

**Fig. 1:**
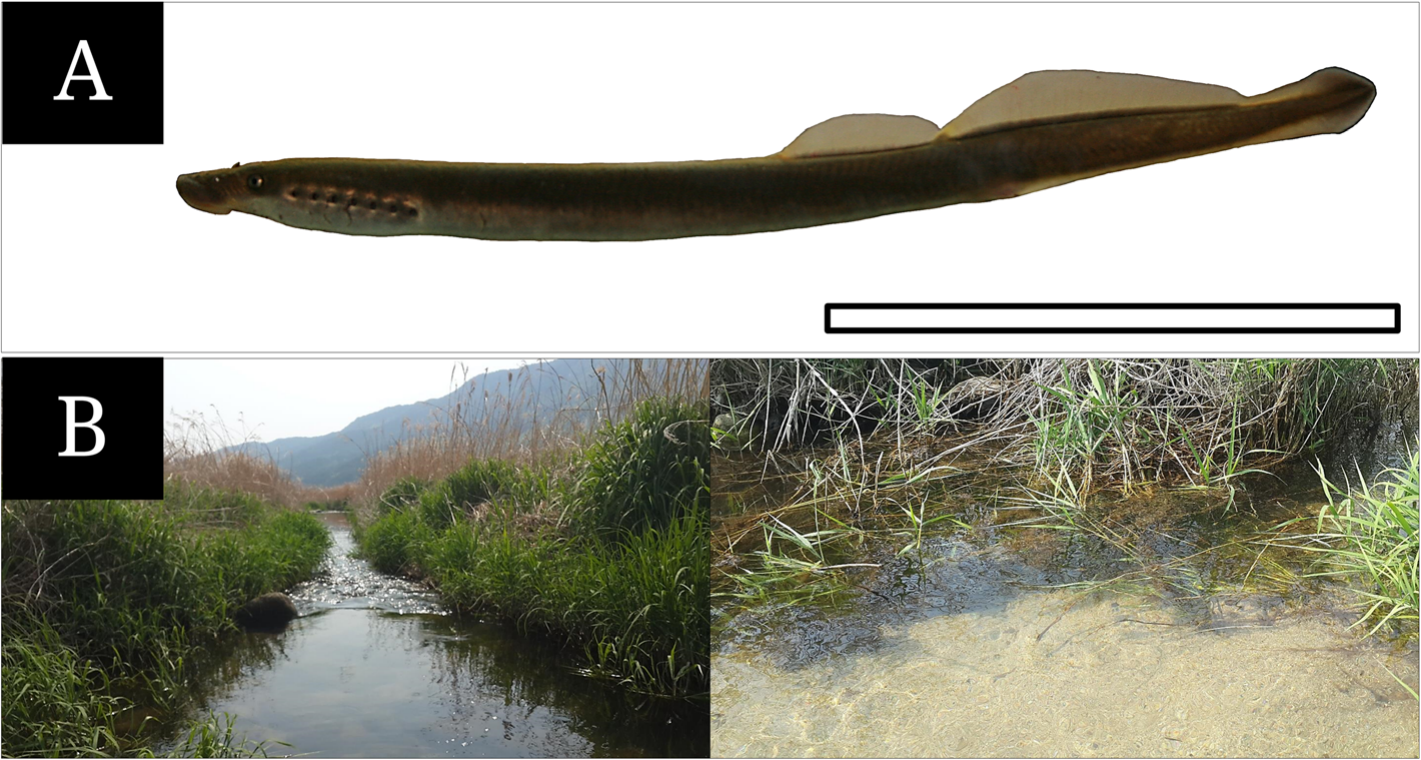
The photographs of adult *Lethenteron reissneri* (**a**) and its habitats (**b**, 35°29’ 40”N, 126°29’ 44”E). The bar indicates 5 cm

Ten adult lamprey *L. reissneri* (124.2 to 166.5 mm in standard length, SL; Fig. 1a) were caught by a scoop net (4 × 4 mm in mesh) in June 2017 in the stream of Daegi-ri, Sandong-myeon, Namwon-si, Jeollabuk-do, South Korea (Fig. 1b). Among the total ten specimens collected, five were fixed in 10% formalin solution buffered at pH 7.4 (0.2 M phosphate buffer) and the rest were taken to the laboratory for two steps of fixing process by 2.5% glutaraldehyde solution (G.A. solution) buffered at pH 7.4 (0.2 M phosphate buffer). For catching the fish, we received a permission allowed by the ministry (a license number: No. 2017–18). All procedures for this study were performed according to the rules under Jeonbuk National University Institutional Animal Care and Use Committee.

### Microscopic investigation

For checking gross structure, the olfactory organ was anatomized using a blade (Feather Safety Razor Co., LTD, Japan) under a stereo microscope (SM; Stemi DV4, Carl Zeiss, Germany), and photographed with a digital camera (TG-3, Olympus, Japan).

A light microscope (LM, Carl Zeiss, Germany) and a scanning electron microscope (SEM, Carl Zeiss, SUPRA40VP, Germany) were used for identifying the cells and the organization of the olfactory epithelium. For histological investigation of the olfactory epithelium, the olfactory tissue was dissected out from the head of the fixed specimens, dehydrated in an ascending ethanol series (50%, 60%, 70%, 80%, 90%, 95%, 100%), cleared in the solution of a one xylene to one 100% ethanol mixed ratio and absolute xylene, and then embedded in paraffin wax (Paraplast, Oxford). 5 μm sections were deparaffinized and then stained with hematoxylin and eosin (H&E) and Masson’s trichrome (Gurr 1956) for general histology. For the surface observation of the olfactory epithelium, the lamellae were dissected out from the head of fixed specimens and fixed again in 2.5% G.A. solution for 24 h. Then the olfactory tissue were fixed in 1% osmium tetroxide solution with 0.1 M phosphate buffer, and dehydrated by a graded series of ethanol, and dried using a t-BuOH freeze dryer (VFD-21S, Vacuum Device Co., Ltd., Ibaragi, Japan). The dried samples were coated with osmium tetroxide by ion sputtering (HPC-1SW, Vacuum Device Inc., Tokyo, Japan) and then observed under the SEM.

## Results

### Anatomy

**Fig. 2:**
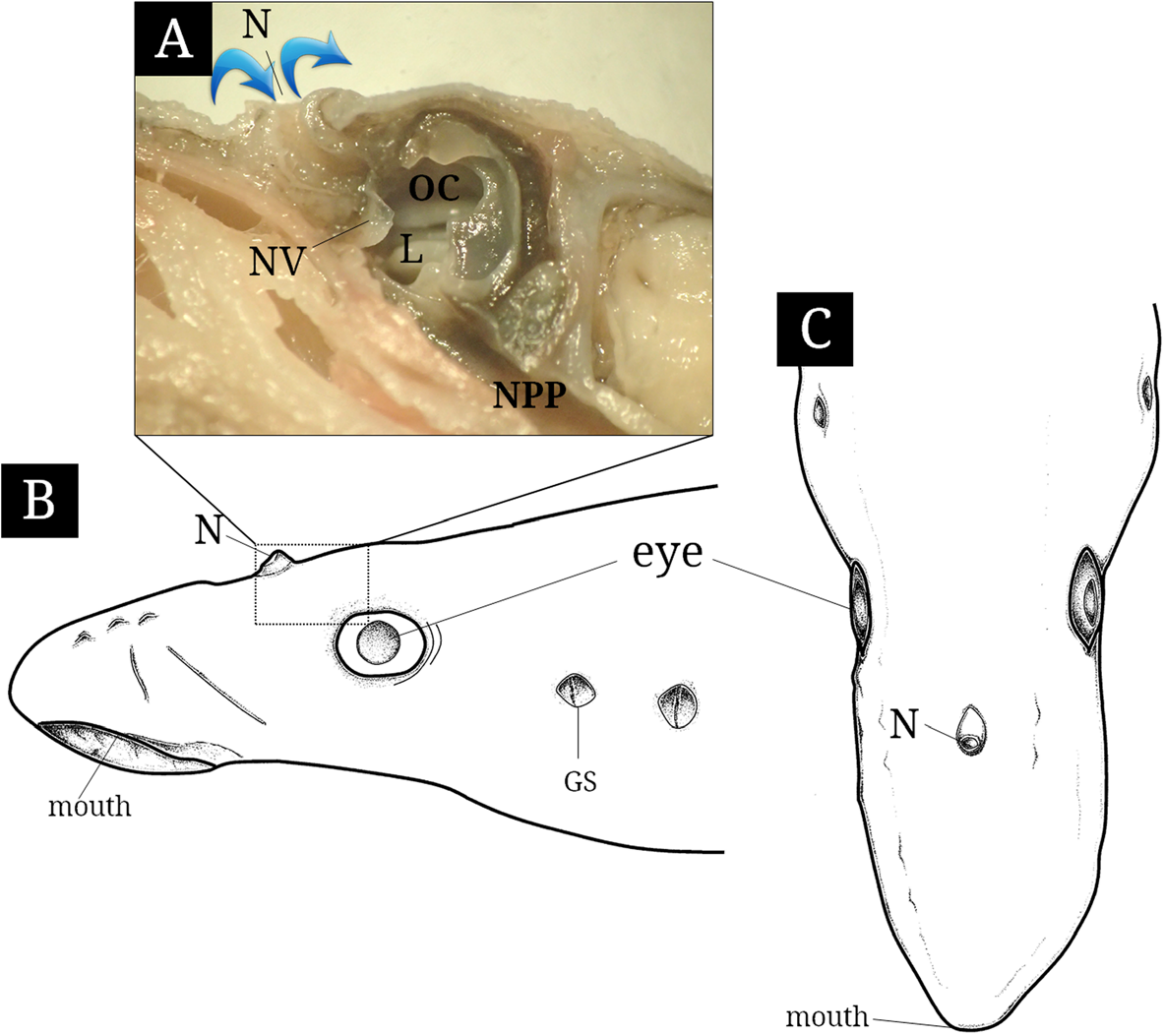
The photograph (**a**) of inner structure and schematic diagrams of lateral (**b**) and upper views of the olfactory organ in *Lethenteron reissneri*. The blue arrows indicate water flowing. GS, external gill slit; L, lamellae; N, nostril; NPP, nasopharyngeal pouch; NV, nasal valve; OC, olfactory chamber

At the top of the head, the single olfactory organ of *L. reissneri* consists of the single nostril, and single olfactory chamber, and single nasopharyngeal pouch (Fig. 2). The single nostril (0.6 to 1.1 mm in major diameter; 0.2 to 0.4 mm in major length) forms a tube shape projecting from the skin surface (Fig. 2b and c). The single olfactory chamber is a gourd-like form and has 19 to 20 lamellae with a longitudinal arrangement on the lateral wall. It is linked proximally with the nasopharyngeal pouch. The nasopharyngeal pouch forms an elongated canal that is expanding toward the caudal direction and closed (Fig. 2a).

### Histology

**Fig. 3:**
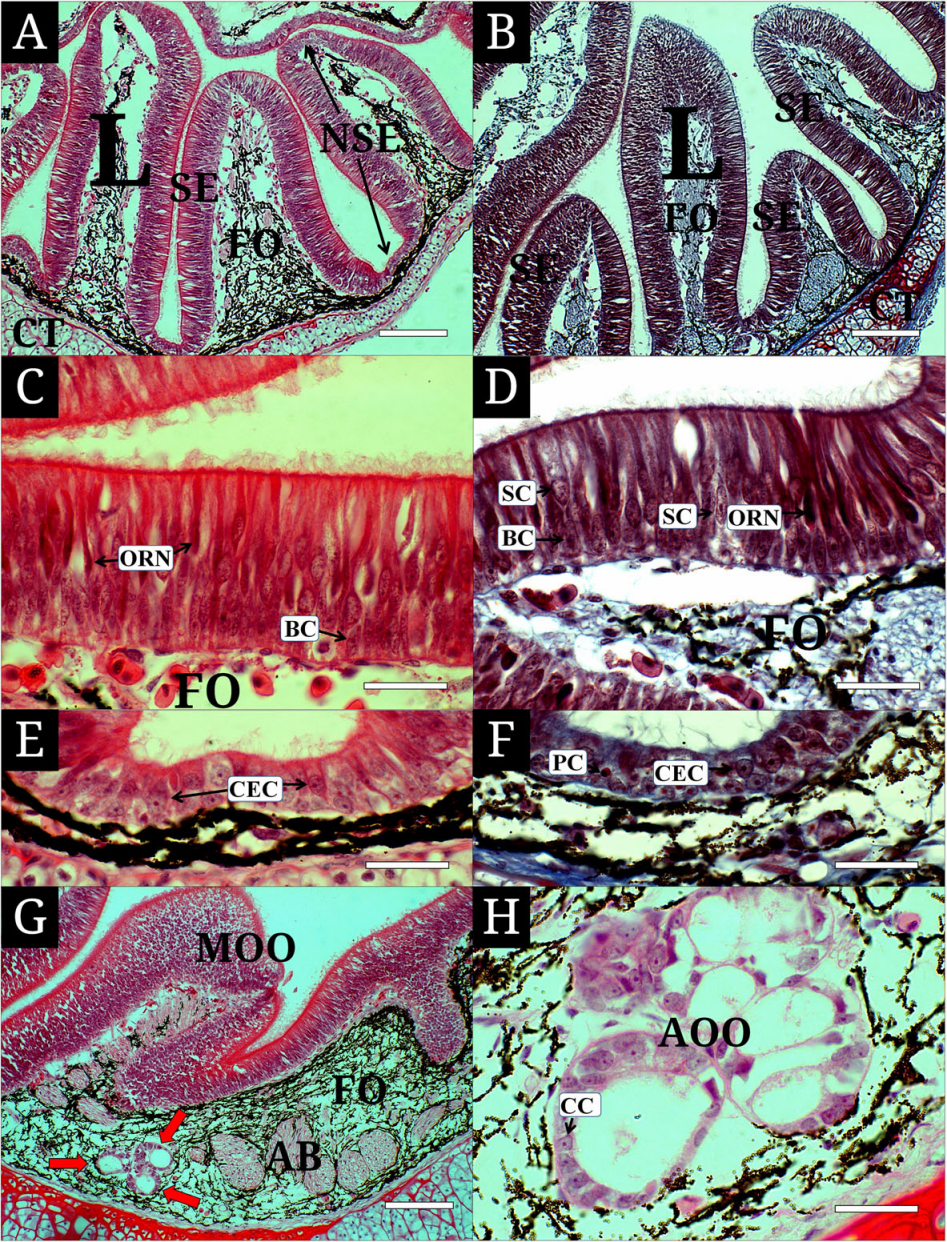
Histological characteristics of the olfactory epithelium of *Lethenteron reissneri*, stained with hematoxylin and eosin (**a, c, e, g**) and Masson’s trichrome. **a, b**), the main olfactory epithelium consisting of the sensory and non-sensory epithelium; **c, d**), the sensory epithelium with olfactory receptor neurons, supporting cells and basal cells; **e, f**), the non-sensory epithelium having columnar epithelial cells and plasma cells; **g, h**), the accessory olfactory organ with olfactory receptor neurons. AOO, accessory olfactory organ; BC, basal cell; CC, cuboidal epithelial cell; CEC, columnar epithelial cell; CT, cartilaginous tissue; FO, fila olfactoria; L, lamellae; MOE, main olfactory epithelium; NSE, non-sensory epithelium; ORN, olfactory receptor neuron; PC, plasma cell; red arrow; accessory olfactory organ; SC, supporting cell; SE, sensory epithelium. Bars indicate 200 μm in **a, b, g** and 50 μm in **c, d, e, f, h**, respectively

In a light micrograph, the olfactory organ of *L. reissneri* is classified into the main olfactory organ (MOO) and accessory olfactory organ (AOO). The MOO contains several lamellae at the lateral wall of the olfactory chamber that is surrounded by the cartilaginous tissue. The lamellae with different size consist of the sensory (SE) and non-sensory (NSE) epitheliums, respectively. The fila olfactoria with abundant axons are present under the epithelial layer (Fig. 3a and b). The SE in both sides of the lamellae shows a continuous type in its distribution. In contrast, the NSE occurs only in the top region of lamellae and in the epithelial region between the lamellae (Fig. 3a and b). The SE of a pseudostratified columnar layer has olfactory receptor neurons (ORN), supporting cells (SC), and basal cells (BC) (Fig. 3c and d). The ORNs are a bipolar cell with two extensions. They show a scarlet nucleus stained with H&E and thick violet with Masson’s trichrome. The SCs are a cylindrical cell, stained more weakly than those of the ORNs with H&E staining and Masson’s trichrome staining, and contains an oval nucleus at the middle part of the epithelium. The BCs are placed vertically at the bottom of the SE. They are absent of any extending branches and has a little cytoplasm. The NSE of a pseudostratified columnar layer is composed of columnar epithelial cells (CEC) and plasma cells (PC) (Fig. 3e and f). The CECs are a large cylindrical cell with numerous cilia at its surface, and show nucleus’s different position arranged to two layers. The large nucleus stains weak scarlet with H&E and has the single prominent nucleolus at the center. The PCs are a small cell and has one-sided nucleus in the cell body. The cytoplasm are stained deep red with Masson’s trichrome.

The AOO is the aggregation of tubular epithelial vesicles that are distributed in fila olfactoria, the connective tissue (Fig. 3g). The epithelium is a simple cuboidal layer that only consists of cuboidal epithelial cells (CC). The CCs have a nucleus with centered nucleolus and little cytoplasm. They are a weak purple stained with H&E (Fig. 3h).

### Ultrastructure of the olfactory epithelial surface

**Fig. 4:**
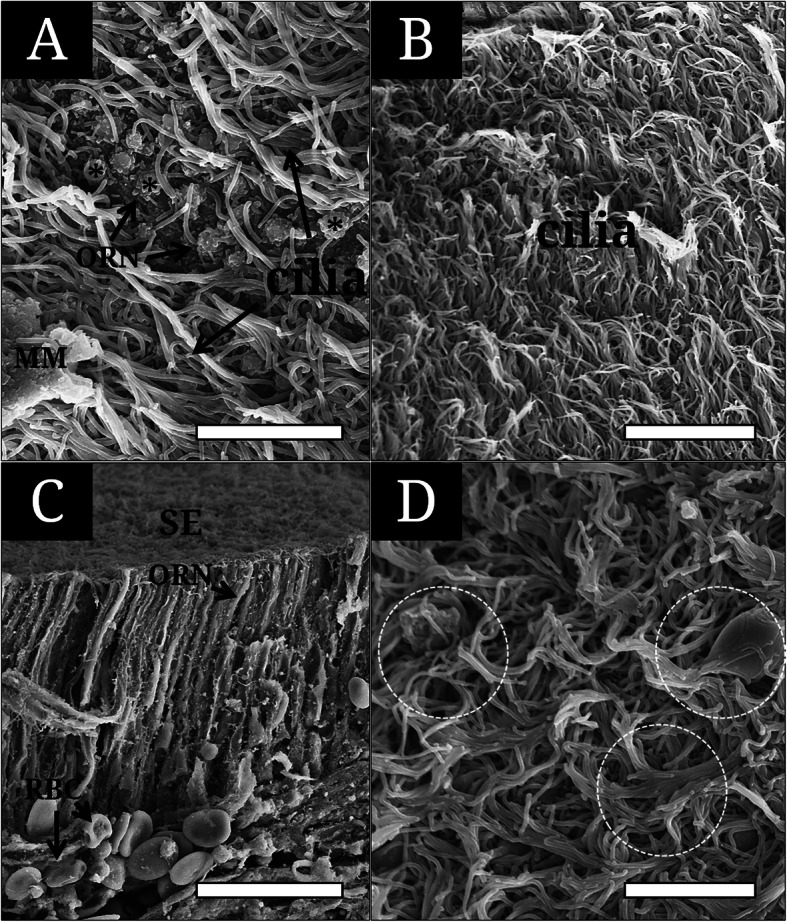
Scanning electron micrographs showing the surface and cross section of the sensory epithelium of *Lethenteron reissneri*. **a**, the sensory epithelium with olfactory receptor neurons, long cilia of the supporting cell and mucous mass; **b**, long cilia covering the sensory surface; **c**, the cross section showing olfactory receptor neurons extending to the surface; **d**, long cilia getting intertwisted and gathered with mucous mass. Asterisk, the ciliated ORN’s knob; broken circle, gathered cilia; MM, mucous mass; ORN, olfactory receptor neuron; RBC, blood cell; SE, sensory epithelium. The bars indicate 5 μm in **a, b, d** and 30 μm in **c**, respectively

The MOO’s epithelia show the three contents: ORN’s knob, SC’s long cilia, and a mucous mass (Fig. 4a and b). The ORN’s knob bears five to eight short cilia on its surface (Fig. 4a) and shows an elongated narrow body extending to the surface as in a light microscopy (Fig. 4c). The length of their cilia is about 0.2–0.5 μm, a quarter of the knob’s diameter (Fig. 4a). Long cilia are intertwined for each other and some are curling with mucus mass (Fig. 4d).

## Discussion

*L. reissneri* shows the unpaired olfactory organ unlike the jawed vertebrates, Osteichthyes and Chondrichthyes, having paired structures at both sides of the snout (Kasumyan 2004). The position of the jawed vertebrate’s olfactory organ was general at the ventral part of the snout in Chondrichthyes and at the dorsal part of the snout in Osteichthyes (Zeiske et al. 1992). Interestingly, however, that of *L. reissneri* is located at the top of the head. Of the same cyclostomata, lamprey has the same position to that of *L. reissneri* (Kleerekoper and Erkel 1960) but hagfishes are above the oral cavity (Theisen 1973, 1976). Such top positions of the olfactory organ in *L. reissneri* as well as in other lampreys may reflect their phylogeny as representative of the lower taxa, not higher taxa such as teleost.

The olfactory organ of *L. reissneri* is characterized by a tube form of single nostril, a single gourd-like chamber, a nasal valve, a nasopharyngeal pouch, a longitudinal arrangement of lamellae, a continuous distribution of the SE, the ciliated SCs, and the AOO, as the same structure in the sea lamprey *Petromyzon marinus* (Kleerekoper and Erkel 1960). Among those characters, a tube form of the nostril of *L. reissneri* are responsible for its habitat, where has a somewhat slow-water currents, being occupied by water plants, gravels and pebbles (Kim and Park 2002; Fig. 1b). The tube form also may be suitable for its ecology that the fish burrows its body into the sand at daytime during the lifetime from the ammocoetes to the adult stage (Kim and Park 2002). Kim et al. (2019) noted that with a contraction and a relaxation of the accessory nasal sac, the tubular nostril helps generate a force to suck water into the olfactory canal. In addition, Cox (2008) opined that the tubular structure assists to reduce thicker “boundary layer” occurring on the snout surface of inactive fishes. The “boundary layer”, generated by a friction when fish swims forward, disturbs to sense external chemical odors and generally is thicker in an inactive fish than fast-swimming fish (Cox 2008). So, tube form on the head of *L. reissneri* seems to be a morphological adaptation and a positional strategy for the reduction of “boundary layer” and effective olfaction under sandy ground.

Meanwhile, *L. reissneri* has 19–20 lamellae in number and are different from the sea lamprey *P. marinus* with 25 (Kleerekoper and Erkel 1960) and other hagfishes, *Myxine glutinosa*, *Eptatretus stoutii* and *E. deani*, with 7 lamellae (Theisen 1976). Generally, in teleost fish, the lamellar number of the olfactory organ is species-specific: absence (*Conidens laticephalus* of the Order Gobiesociformes, *Oryzias latipes* and *O. sinensis* of the Beloniformes, and *Periophthalmus modestus*, *P. magnuspinnatus* of the Perciformes), only one (*Hemiramphus sajori*, *Cheilopogon agoo*, and *Cololabis saira* of the order Atheriniformes), up to 120 (*Conger myriaster* of Anguilliformes) and 230 lamellae (*Holopagus guentheri* of Perciformes) (Yamamoto 1982; Kasumyan 2004; Kim et al. 2019). It was known that the increase of lamellae leads to the increase of distributional area of olfactory receptor neurons (Kasumyan 2004). Teichmann (1954) also documented that microsmatic catfishes with greater lamellar number show more active response to any chemical stimulant than one with lesser lamellae. At least, the greater number in lamellae of *L. reissneri* may be more likely to have higher olfactory dependence than other cyclostomata hagfishes with less.

In fish’s olfactory epithelium, it is common that the SE is a simple pseudostratified layer with numerous neurons and the NSE is a stratified squamous layer consisting of stratified epithelial cells (Hara 1986). Interestingly, the SE and NSE of *L. reissneri* consist of a simple pseudostratified layer, first findings, not reporting in other cyclostomatas and *P. marinus*. Almost jawed fishes has shown the NSE formed from a stratified squamous layer which has various-shaped cells from columnar or cuboidal types in deeper layer to squamous or flattened types at the surface (Hara 1986; Ghosh and Chakrabarti 2009; Kim et al. 2018, 2019). In contrast, as the NSE of *L. reissneri* is a histological organization unlike those of the above higher taxa, it may be considered a specific histological character of lamprey belonging to the cyclostomata.

In jawed fishes, the length of olfactory sensory cilia varies by species: 2-3 μm in *Daniro rerio* (Hansen and Eckart 1998), the maximum 10 μm in *Acipenser baeri* (Hansen and Zielinski 2005), 10-30 μm similarly in *Lota lota* L. (Gemne and Døving 1969) and *Calamoichthys calabaricus* (Schulte and Holl 1971). Further, lampreys show a different length in related congeneric species: 0.2–0.5 μm in *L. reissneri* (this study) vs. 5–6 μm in *L. fluviatilis* (Thornhill 1967). Menco (1980) reported that in a comparative study of frog, ox, rat, and dog using a freeze-fracturing the effectiveness of ciliary mechanism to sense external odors is related to the increase of cilia area by number, length, and any attaching component. Twisted ciliary aggregations become packed mat within mucus and then may help make mucus film covering the olfactory surface (Farbman 1992).

Consequently, the anatomy and histology of the olfactory organ of *L. reissneri* may well reflect its microhabitat where water flows slowly and bottom structure consists of sand, gravels and pebbles, and its ecological habit that fish burrows its body into sandy ground. Among the characters, both 19–20 lamellae and the ORN’s short ciliary length are at least regarded as a useful taxonomic character for lamprey.

## Conclusion

The Far Eastern brook lamprey *Lethenteron reissneri*, the non-parasitic and non-anadromous fish, prefers to live in slow-flowing water in a river or mid-stream, and its adult is a nocturnal fish that hides beneath sand, gravels or pebbles at daytime and act at night. A single gourd-like chamber, a nasal valve, a nasopharyngeal pouch, a longitudinal arrangement of lamellae, and the AOO are general features responsible for primitive vertebrate taxa and may be a morphological adaption for effective sensory activity in such habitat. In particular, 19 to 20 lamellae and a quarter ciliary length (0.2–0.5 μm) of the knob diameter in the ORN may be a useful taxonomic character compared to the sea lamprey *P. marinus*, and both the SE and NSE of a simple pseudostratified layer may be regarded as a unique histological character, not reported in other cyclostomata.

## Data Availability

Not applicable.
